# Status Migrainosus and Endometriosis: A Case Report and Review of the Literature

**DOI:** 10.7759/cureus.19621

**Published:** 2021-11-16

**Authors:** Lexi R Frankel, Richard Medina, Michael Ashley, Jose L Batista, Livasky Concepion

**Affiliations:** 1 College of Allopathic Medicine, Nova Southeastern University Dr. Kiran C. Patel College of Allopathic Medicine, Davie, USA; 2 Internal Medicine, Aventura Hospital and Medical Center, Aventura, USA; 3 Medicine, Aventura Hospital and Medical Center, Aventura, USA; 4 Graduate Medical Education (GME), Aventura Hospital and Medical Center, Aventura, USA

**Keywords:** endometriosis, factor ii mutation, migraine, migraine with aura, status migrainosus

## Abstract

Status migrainosus is a migraine complication describing an attack lasting longer than 72 hours. In this paper, we present a case of a 34-year-old female with a history of severe endometriosis and hypercoagulable factor type II disease who presented to the emergency department (ED) with a three-week history of new-onset intractable migraine with aura. Imaging findings revealed a frontal T2/FLAIR hyperintensity, venous anomaly, and bilateral optic nerve thickening. The patient was admitted for three days of inpatient treatment with improvement of her symptoms.

## Introduction

Migraine is a highly debilitating disorder, affecting up to 12% of the population annually. Status migrainosus, or intractable migraine, is a complication of migraine describing an attack lasting longer than 72 hours [1]. There is a strong genetic component to migraine, and its incidence is further increased among women and patients with endometriosis and hypercoagulable disorders [2,3]. The diagnosis of migraine has been linked to increased risk of stroke, multiple sclerosis, cardiovascular disorders, and psychiatric disorders [4-6].

Migraine with aura is described as migraine headache with reversible transient focal neurological symptoms developing from the cortex or brainstem. While the vast majority of auras are visual, symptoms may also include sensory, motor, retinal, brainstem, or speech disturbances. Migraine with aura is associated with a twofold increase in ischemic stroke risk when compared with migraine without aura [7]. As the presentation of migraine with aura is often nonspecific and mimics other disorders, its diagnosis and workup are complicated. In the presence of other comorbidities, the diagnosis of complex migraine becomes challenging and often requires imaging to rule out cerebral-vascular accidents and other pathologies associated with significant morbidity and mortality.

## Case presentation

**Figure 1:**
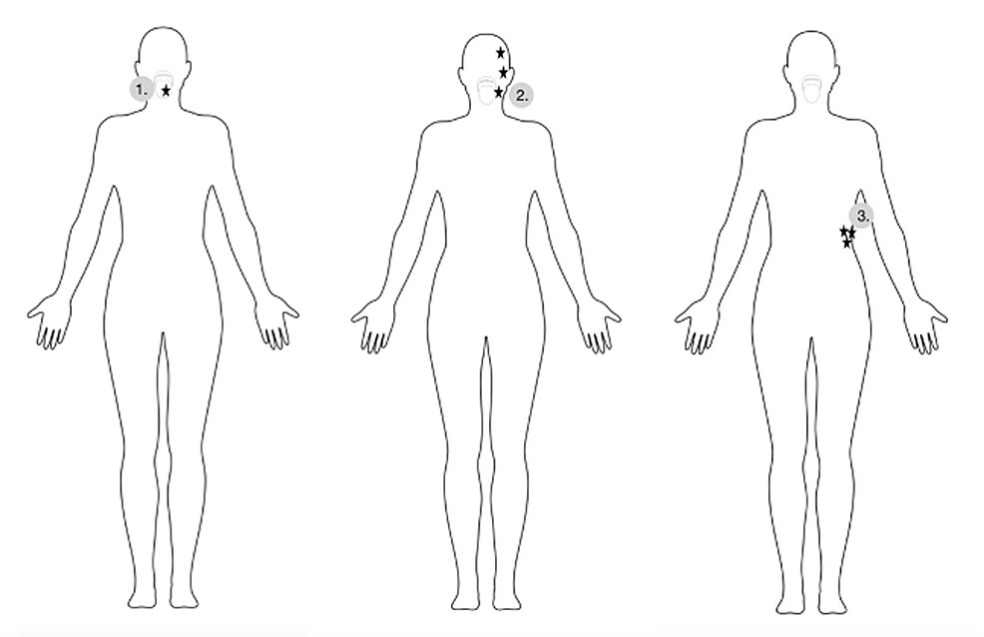
Visual depiction of the patient’s migrating sensory aura (black stars) from the tongue (1) to the left side of the face (2) to the left flank (3).

**Figure 2:**
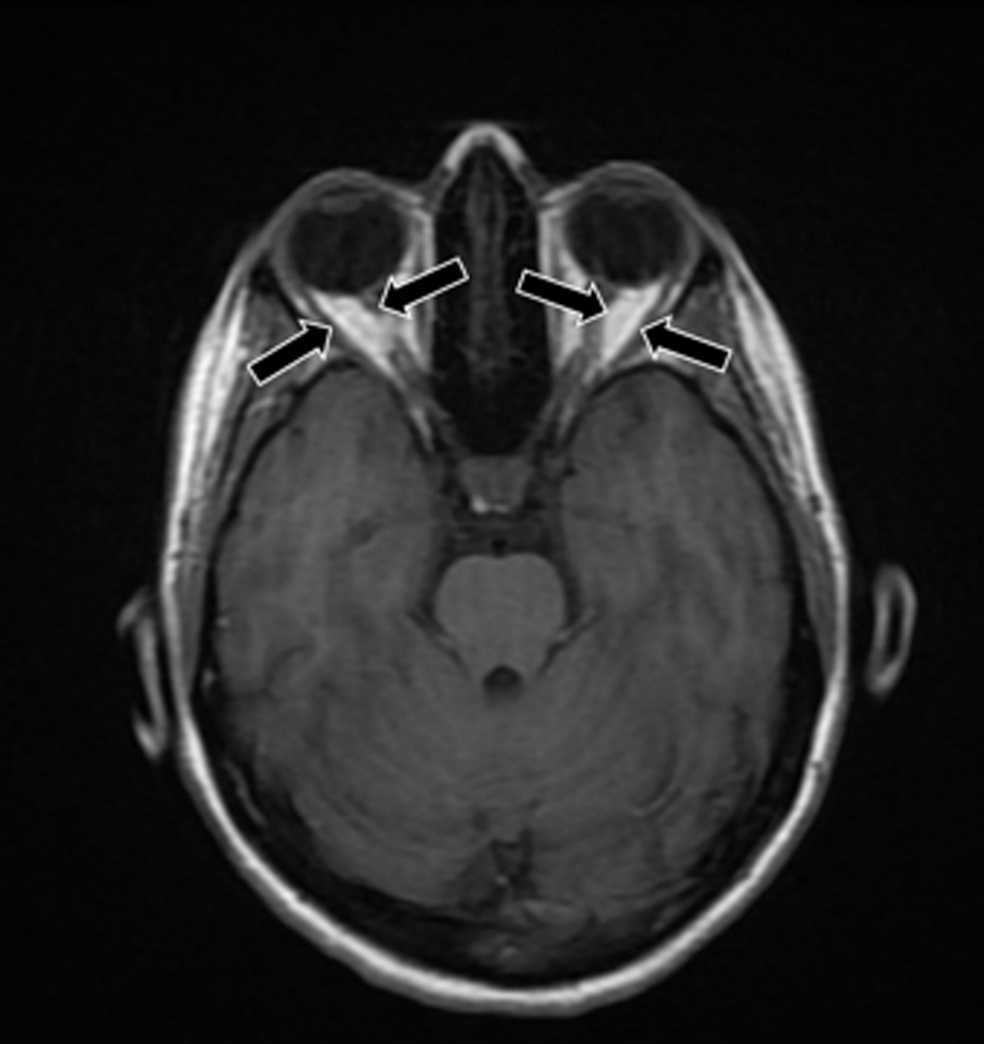
Brain CT revealing mild thickening of the bilateral optic nerves (black arrows).

**Figure 3:**
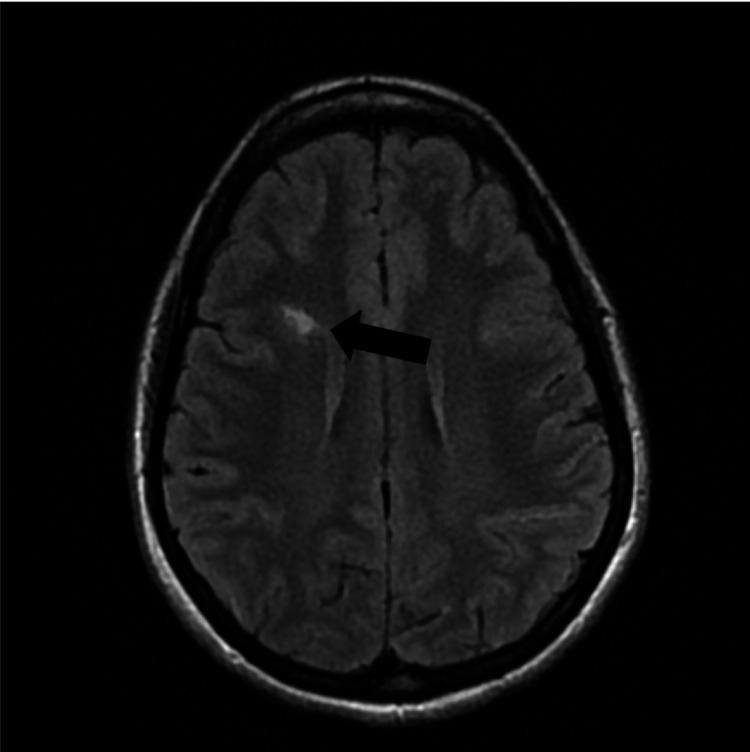
Brain MRI demonstrating a 1 cm right frontal subcortical bright T2/FLAIR hyperintensity without evidence of abnormal enhancement (black arrow).

**Figure 4:**
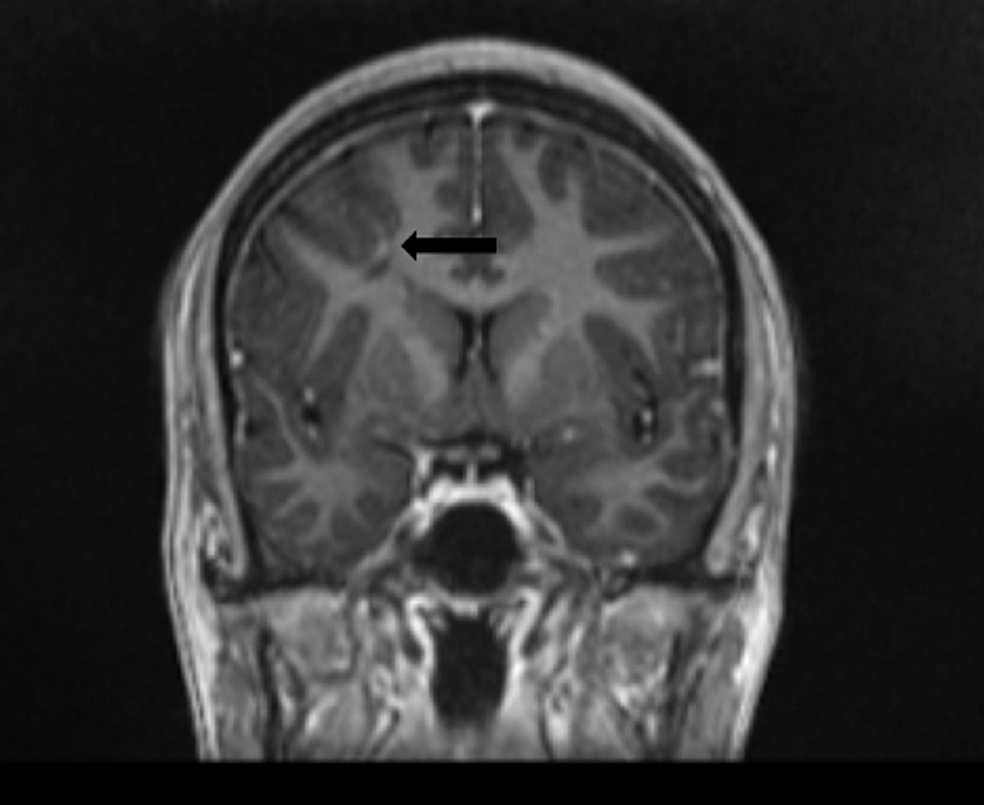
Brain MRI demonstrating a small adjacent developmental venous anomaly (black arrow) adjacent to the right frontal subcortical bright T2/FLAIR hyperintensity.

In this case, we present a 34-year-old female with a past medical history of endometriosis, factor II hypercoagulable disorder, and menstrual migraine without aura who presented with three weeks of intractable migraine with neurological features. She described a bilateral squeezing frontal headache that remained at a “7/10” pain severity for three weeks prior to arrival. One week before she presented, she began experiencing visual disturbances consistent with a scintillating scotoma that lasted for about two to three hours every day. A few days later, she began experiencing tongue numbness and left face paresthesia that migrated to various locations of her face followed by her left flank (Figure 1). The next day, she was evaluated by a neurologist via a telehealth visit and was subsequently diagnosed with intractable complex migraine with aura and prescribed methylprednisolone and ubrogepant (calcitonin gene-related peptide receptor antagonist) 100 mg as a rescue treatment. However, the following morning, she woke up with anomic dysphasia and another episode of tongue numbness, after which she presented to the emergency department (ED) for evaluation. She did not take her prescribed medications from her telehealth visit, including ubrogepant and methylprednisolone, prior to arrival. In the ED, she endorsed a headache, nausea, dizziness on standing, photophobia, and resolution of her paresthesias. Physical examination was grossly intact with preservation of motor strength, sensation, and cranial nerves. On analysis of complete blood count (CBC) and comprehensive metabolic panel (CMP), a decreased hemoglobin of 10.6 g/dL with a mean corpuscular volume of 73.6 fl consistent with microcytic anemia was the only abnormal finding. This finding was consistent with this patient’s history of iron deficiency anemia secondary to heavy menstrual bleeding. Head computed tomography angiography (CTA) revealed mild thickening of the bilateral optic nerves (Figure 2). Brain magnetic resonance imaging (MRI) demonstrated a nonspecific 1 cm right frontal subcortical bright T2/FLAIR hyperintensity (Figure 3) and a small adjacent developmental venous anomaly without evidence of thrombosis (Figure 4). Although unlikely, early thrombosis could not be ruled out. Subsequent chest X-ray, neck CTA, head MRA, neck MRA, and orbit MRI were obtained, all of which were unremarkable.

After several hours, her headache did not improve, and she was admitted to the floor for further management. The patient was treated with butalbital/acetaminophen/caffeine 50 mg/325 mg/40 mg once, ketorolac 15 mg IV once, dexamethasone 6 mg PO once, and metoclopramide 10 mg IV once. Neurology was consulted. The following morning, the patient refused the prescribed medications for fear of triggering increased pain or another neurological disturbance. She was counseled on the importance of compliance and took her prescribed medications as directed. Neurology began a three-day course of methylprednisolone 1 g daily, topiramate 25 mg three times a day, and naproxen 500 mg twice a day. The condition of the patient improved, and she was discharged with significant improvement of her symptoms. She was instructed to continue taking topiramate 25 mg three times daily, naproxen 500 mg two times daily as needed, and ubrogepant 100 mg as needed for migraine rescue treatment.

## Discussion

To our knowledge, this is the first case report of a patient with worsening endometriosis presenting with new-onset migraine with aura. The patient presented in this report had a 10-year history of stage IV endometriosis, requiring five endometriotic implant ablation surgeries and two endometrioma resections. This patient had a pelvic MRI in 2019 that showed growth of uterine and ovarian lesions compared with a previous MRI. A gynecological examination by her OB/GYN in 2019 also demonstrated growth of ovarian and vaginal implants. At this time, two years prior to admission, the patient’s OB/GYN recommended surgery for her growing lesions, which she deferred for fear of being in the hospital during the COVID-19 pandemic. Additionally, this patient’s history of type II hypercoagulable disease prevented her from taking oral contraceptive pills (OCPs) to control endometriosis symptoms and growth. The incidence of migraine has been previously associated with endometriosis [8,9]. The use of estrogen-containing OCPs in patients suffering from migraine with aura is contraindicated due to the risk of increased vasodilation, migraine frequency, and stroke [10]. As a result, this patient was unable to take OCPs for endometriosis. While GnRHa are an option for patients with severe endometriosis, this patient had already been treated three years prior with a GnRHa for six months. She deferred further GnRHa therapy as a result of side effects. This patient’s worsening endometriosis over the past two years combined with her deferral of surgery and GnRHa therapy and inability to take OCPs likely contributed to her late-onset development of complex migraine. New-onset complex migraine in a woman of childbearing age may warrant further investigation into whether the patient has undiagnosed or worsening endometriosis.

Hypercoagulable diseases and states have also been implicated in the development of migraine [3]. The most currently accepted theory of the link between migraine and hypercoagulability is that ischemia caused by microemboli leads to cortical spreading depression in migraineurs, often seen as a migrating aura [3]. In this patient, cortical spreading depression may have been responsible for the migration of her aura from her tongue to several areas of her face and to her flank. The patient in this report suffered from hypercoagulable factor II disease, further supporting the microembolic pathophysiology of her disease progression. As the link between clotting and migraines has become more clear in recent years, some studies have even questioned screening all women suffering from migraine with aura for biological thrombophilia [11].

Imaging patients undergoing migraine is another diagnostic challenge. The findings on brain MRI may include T2/FLAIR hyperintensities, representing microemboli [12]. However, as this finding is nonspecific and it may correlate with inflammatory processes such as infection or multiple sclerosis, further monitoring and clinical correlation are warranted. The nonspecific 1 cm right frontal subcortical bright T2/FLAIR hyperintensity on this patient’s MRI likely signified microembolic changes secondary to years of menstrual migraine. Although this finding was not redemonstrated on orbit or brain MRI, this patient’s head CTA demonstrated bilateral mild thickening of the optic nerves. In the setting of this patient’s baseline hypercoagulable state, this thickening may be the result of prolonged ischemia, swelling, or thickening of the optic nerves, which has been correlated with migraine with visual aura [13]. This imaging finding may explain this patient’s scintillating scotoma. This patient’s MRI also demonstrated a small venous anomaly (Figure 4). Previous studies have noted a possible correlation between such venous anomalies and migraine; however, the majority of these studies are based on case reports and retrospective data [14]. This venous anomaly was also an important source of migraine to consider in this patient that further complicated the etiology of her disease.

This patient’s initial refusal to take her medications as directed is not an uncommon phenomenon among migraineurs. In fact, one study noted the increased prevalence of cephalalgiaphobia, the fear of headache attack or headache worsening, in patients suffering from migraine [15]. As a result of fear of migraine attacks, patients often take prophylactic medication without necessity or develop food or medication aversion. Our case highlights the prevalence of cephalalgiaphobia in patients with migraine and recognizes the necessity of early recognition of this phenomenon for early treatment of migraine attacks in the acute setting. Another barrier that may have prevented proper care in this patient was the telehealth approach of her neurology appointment prior to presentation. This type of visit may have prevented a full physical examination, which could have precluded proper treatment.

The limitations of this case report are important factors to consider. Importantly, this report represents the findings of a single patient and therefore may not be widely applicable. While this patient presented with new-onset migraine with aura in the setting of worsening endometriosis, causation cannot be assumed from this one report. For this reason, we suggest that further studies be conducted on a larger scale to assess the correlation between these variables.

## Conclusions

While endometriosis and chronic migraine have been linked previously in the literature, this is the first case report to discuss worsening endometriosis in a patient with new-onset status migrainosus. Status migrainosus and migraine with aura, especially in the setting of a history of hypercoagulable disease and endometriosis, is a diagnostic challenge requiring extensive workup and imaging. For women with status migrainosus, we suggest an investigation into gynecological history to assess for history or symptoms of endometriosis. Further research on the association between new-onset migraine with aura and endometriosis is suggested to prevent and treat this debilitating condition.
